# A Systematic Approach to Identify and Characterize the Effectiveness and Safety of Novel Probiotic Strains to Control Foodborne Pathogens

**DOI:** 10.3389/fmicb.2019.01108

**Published:** 2019-05-17

**Authors:** Diana I. Ayala, Peter W. Cook, Jorge G. Franco, Marie Bugarel, Kameswara R. Kottapalli, Guy H. Loneragan, Mindy M. Brashears, Kendra K. Nightingale

**Affiliations:** ^1^International Center for Food Industry Excellence, Department of Animal and Food Sciences, Texas Tech University, Lubbock, TX, United States; ^2^Influenza Division, National Center for Immunization and Respiratory Diseases, Centers for Disease Control and Prevention, Atlanta, GA, United States; ^3^Center for Biotechnology and Genomics, Texas Tech University, Lubbock, TX, United States

**Keywords:** lactic acid bacteria, probiotics, characterization, safety, pathogens

## Abstract

A total of 44 lactic acid bacteria (LAB) strains originally isolated from cattle feces and different food sources were screened for their potential probiotic features. The antimicrobial activity of all isolates was tested by well-diffusion assay and competitive exclusion on broth against *Salmonella* Montevideo, *Escherichia coli* O157:H7 and *Listeria monocytogenes* strain N1-002. Thirty-eight LAB strains showed antagonistic effect against at least one of the pathogens tested in this study. Improved inhibitory effect was observed against *L. monocytogenes* with zones of inhibition up to 24 mm when LAB overnight cultures were used, and up to 21 mm when cell-free filtrates were used. For *E. coli* O157:H7 and *Salmonella* maximum inhibitions of 12 and 11.5 mm were observed, respectively. On broth, 43 strains reduced *L. monocytogenes* up to 9.06 log_10_ CFU/ml, 41 reduced *E. coli* O157:H7 up to 0.84 log_10_ CFU/ml, and 32 reduced *Salmonella* up to 0.94 log_10_ CFU/ml 24 h after co-inoculation. Twenty-eight LAB isolates that exhibited the highest inhibitory effect among pathogens were further analyzed to determine their antimicrobial resistance profile, adhesion potential, and cytotoxicity to Caco-2 cells. All LAB strains tested were susceptible to ampicillin, linezolid, and penicillin. Twenty-six were able to adhere to Caco-2 cells, five were classified as highly adhesive with > 40 bacterial cells/Caco-2 cells. Low cytotoxicity percentages were observed for the candidate LAB strains with values ranging from -5 to 8%. Genotypic identification by whole genome sequencing confirmed all as members of the LAB group; *Enterococcus* was the genus most frequently isolated with 21 isolates, followed by *Pediococcus* with 4, and *Lactobacillus* with 3. In this study, a systematic approach was used for the improved identification of novel LAB strains able to exert antagonistic effect against important foodborne pathogens. Our findings suggest that the selected panel of LAB probiotic strains can be used as biocontrol cultures to inhibit and/or reduce the growth of *L. monocytogenes, Salmonella*, and *E. coli* O157:H7 in different matrices, and environments.

## Introduction

In the United States (U.S.), an estimated 9.4 million cases of foodborne illness, 55,961 hospitalizations, and 1,351 deaths per annum are attributed to 31 identifiable foodborne pathogens (Scallan et al., 2011). Ninety-five percent of the total illnesses, hospitalizations, and deaths were estimated to be caused by only 15 pathogens, including *Listeria monocytogenes*, non-typhoidal *Salmonella* and *Escherichia coli* O157:H7 (Hoffmann et al., 2015). Non-typhoidal salmonellosis is a leading cause of bacterial gastroenteritis in the U.S. and worldwide and foodborne illnesses caused by *L. monocytogenes* and *E. coli* O157:H7 are associated with exceptionally high morbidity and mortality rates (Scallan et al., 2011). The growing concern of antimicrobial resistance (AMR) coupled with the increased demand for a safe food supply by consumers has prompted an increased interest in the use of probiotics as a natural biocontrol strategy to reduce foodborne pathogens along the food continuum.

Probiotics are live, naturally occurring microorganisms that in adequate amounts confer benefits to the host (Fuller, 1992). Probiotics have also emerged as a natural alternative to antimicrobials in animal feed to promote animal health [also referred to as direct fed microbials (DFMs) in animal feed] and chemical interventions to control foodborne pathogens in human and pet food. Modes of action used by probiotics include production of antimicrobial compounds (i.e., bacteriocins and organic acids) and competitive exclusion. Probiotic strains compete with pathogens for nutrients and minerals as well as receptors or adhesion sites in the host intestinal tract, therefore displacing pathogen adhesion to host intestinal epithelial cells. Probiotics also improve host intestinal barrier function and activate mucosal immunity (McAllister et al., 2011). Together these modes of probiotic action and stimulation of the host immune system, interfere with the pathogens’ essential cell functions causing leakage of cytoplasmic components and cytotoxicity, thus leading to pathogen cell death (Yirga, 2015).

Due to their demonstrated antagonistic effects against foodborne and spoilage bacteria, the probiotic strains most commonly used to promote host health and control foodborne pathogens are lactic acid bacteria (LAB) from the genera of *Lactobacillus* and *Enterococcus* (Imperial and Ibana, 2016). LAB are an order of gram-positive, non-spore forming cocci, bacilli or rods that are generally non-respiratory and lack catalase; they are able to ferment glucose to produce lactic acid or lactic acid, CO_2_ and ethanol. Most LAB are beneficial to the host; however, some LAB are pathogenic or opportunistic pathogens to animals and humans (e.g., some *Streptococcus* and *Enterococcus* spp.) and careful selection criteria should be evaluated in selecting probiotic strains to be included as DFMs in animal feed and probiotics in human and pet food (Yirga, 2015). LAB are ubiquitous in nature and can be routinely isolated from vegetation and a wide range of raw foods including milk and milk products, meat, and produce (Mohania et al., 2008; Quinto et al., 2014). Additionally, LAB are natural commensals of the gastrointestinal tract (GIT) of mammals, they constitute the dominant indigenous lactic microbiota present, this enables LAB to beneficially affect the host by an improvement of the microbial profile in the gut (Fuller, 1997; Brashears et al., 2003).

The criteria and safety assessment to select new probiotic strains to be used as a biocontrol intervention includes identification and characterization of non-pathogenic strains with antagonistic features against pathogens in a host or other systems where pathogen control is needed. Desirable features of a potential probiotic strain include (i) attach to and colonize intestinal epithelial cells, (ii) exhibit susceptibility to antibiotics, (iii) stably survive and have metabolic activity in the small intestine, and (iv) remain viable during delivery (Krehbiel et al., 2003; Gaggìa et al., 2010; Sanders et al., 2010; Seo et al., 2010). Benefits of supplementing animal feed and pet food with probiotics include, (i) improve resistance to disease by a beneficial shift in the microbial community, (ii) reduce pathogen colonization, (iii) stimulate host immunity and (iv) overall improved host health (Beauchemin et al., 2003; Quigley, 2011). LAB include at least 13 genera where thousands of genetically diverse strains differ in their ability to benefit the host through controlling pathogens and improving overall host health (Liu et al., 2014).

Previous findings have shown that effectiveness to control enteric pathogens and health benefits conferred by probiotics depend on strain-specificity, with different results for adhesion, autoaggregation, and immunomodulatory effect depending on the strain used (Santosa et al., 2006; Angelakis et al., 2011). The overall aim of this study was to use a combination of genotypic and phenotypic assays to characterize a set of novel LAB strains for their ability to control *Salmonella, E. coli* O157:H7 and *L. monocytogenes* throughout the food continuum, including pre-harvest applications in animal feed and post-harvest applications in pet and human food along with environments associated with pet and human food processing and handling. A collection of 44 LAB strains from cattle feces and human food were characterized by agar well-diffusion, competitive broth exclusion assays (to identify LAB strains with antagonistic effects against *L. monocytogenes, Salmonella*, and *E. coli* O157:H7), whole genome sequencing (for taxonomic identification and to predict bacteriocin and virulence gene carriage), antimicrobial susceptibility, and cell culture assays (to determine adhesion to and cytotoxicity against intestinal epithelial cells).

## Materials and Methods

### Bacterial Strains and Growth Conditions

A total of 53 LAB isolates (out of an initial set of > 200 novel strains) from cattle feces and different food sources including meat, fruits, and vegetables that showed initial antagonistic activity against *L. monocytogenes, Salmonella*, and *E. coli* O157:H7 were obtained from the stock culture collection of the International Center of Food Industry Excellence at Texas Tech University (ICFIE: TTU). Isolates were streaked for isolation onto de Man Rogosa and Sharpe (MRS) agar plates (Merck, Darmstadt, Germany), and incubated aerobically at 37°C for 48 h to obtain well-isolated colonies. A single colony was selected and grown in 9-ml of fresh MRS broth (Criterion, Hardy Diagnostics, CA, United States) at 37°C for 12–18 h. Nine of the 53 LAB strains did not further grow in MRS broth and were removed from the study. Pure cultures of 44 remaining LAB strains were grown up in MRS broth as described above, preserved on cryobeads (Key Scientific, Stamford, TX, United States) and stored at -80°C until further use. Foodborne pathogen isolates including *L. monocytogenes, Salmonella* and *E. coli* O157:H7 were streaked onto Tryptic Soy Agar (TSA, Becton, Dickinson, Le Pont de Chaix, France) and incubated at 37°C for 18–24 h. A single colony of each pathogen was selected and grown individually in 9-ml of Brain Heart Infusion (BHI) broth (Merck, Darmstadt, Germany). Overnight cultures of LAB and pathogenic strains were used to perform agar-well diffusion and competitive exclusion assays as detailed below to determine the antagonistic activity of all 44 LAB strains against each pathogen.

### Agar-Well Diffusion Assay

This set of experiments was performed based on the method described previously by Vinderola et al. (2008) with slight modifications. Overnight cultures (incubated in nutrient rich media for 12–18 h at 37^o^C) of foodborne pathogens were diluted to achieve a concentration of 10^5^ CFU/mL, swabbed onto BHI plates and incubated at 37^o^C for 24 h to create a lawn. To assay the antagonistic activity of each LAB strain against each foodborne pathogen, five 6-mm wide wells cut into a BHI agar plate were filled with a 100 μl aliquot of the following: (i) 10^8^ CFU/mL of each LAB strain overnight culture in duplicate wells, (ii) cell-free filtrate (CFF) of LAB overnight cultures, passed through a sterile 0.45 μM filter (Sigma-Aldrich, St. Louis, MO, United States), in duplicate wells, and (iii) MRS broth (control) in a single well. Plates were first incubated at 4°C for 2 h to allow suspensions to diffuse in the agar followed by incubation for 24 h at 37°C (Zavisic et al., 2012). Antagonistic activity was determined by the development of clear zones of inhibition around each well and measured using a caliper. Overall scores of inhibition were calculated by summing up values observed for overnight cultures and CFF across all three pathogens, and potential LAB probiotic strains were ranked for antagonistic activity against all three foodborne pathogens.

### Competitive Exclusion Broth Culture Assay

Overnight cultures of each pathogen and LAB strains were prepared as described above for agar well diffusion assays and co-inoculated at 10^5^ and 10^6^ CFU/mL, respectively in Tryptic Soy Broth (TSB; Oxoid Ltd., Basingstoke, United Kingdom) supplemented with 1 g l^-1^ Tween 80 (Acros, Organics, NJ, United States) and incubated at 37°C with slight agitation (130 rpm). A previous study demonstrated that addition of tween to TSB allows growth of both LAB and gram-negative pathogens, including *E. coli* O157:H7 and *Salmonella* (Cálix-Lara et al., 2012). Samples were diluted and plated onto Modified Oxford Agar (MOX; Becton, Dickinson and Company), Xylose Lysine Tergitol 4 Agar (XLT4; Becton, Dickinson and Company), MacConkey agar with sorbitol (SMAC; Criterion, Hardy Diagnostics, CA, United States), and MRS agar plates to enumerate *L. monocytogenes, Salmonella, E. coli* O157:H7, and LAB strains, respectively, at 0, 6, 12, and 24 h of co-inoculation. MOX, XLT4, and SMAC plates were incubated at 37°C for 24 h, and MRS plates were incubated at 37°C for 48 h. Antagonistic activity of each LAB strain was determined by pathogenic reduction with respect to control samples (pathogen cultures without LAB) at each time point. As for agar well diffusion assays, pathogenic reductions were summed up across all time points and across all three pathogens, and potential LAB probiotic strains were ranked based on their antagonistic effect. The top 20 ranking LAB strains from the agar well diffusion and competitive exclusion broth assays (*n* = 28 LAB strains in total) were selected for further phenotypic and genotypic evaluation by the assays below.

### Caco-2 Cell Attachment Assay

The human epithelial intestinal cell line Caco-2 was used to evaluate the *in vitro* ability of the top ranking LAB strains to adhere to the GIT. Caco-2 cells (ATCC HTB-37^TM^) were maintained in Eagle’s Minimum Essential Medium (EMEM, ATCC 30-2003^TM^) supplemented with 20% fetal bovine serum (FBS, ATCC 30-2020^TM^) and 1% of penicillin/streptomycin solution (10,000 U/mL of penicillin and 10,000 μg/mL streptomycin) (PenStrep) (Gibco, Thermo Fisher Scientific) at 37°C in a water-jacketed incubator with 5% CO_2_. Three days before attachment assays were performed, Caco-2 cells were seeded into 24-well tissue culture plates at a target density of 5 × 10^4^ cells/well to achieve a confluent density of 1 × 10^5^ cells/well in EMEM supplemented with 20% FBS and 1% PenStrep. For each attachment assay, cell culture media (1 mL/well) was removed and replaced with antibiotic-free EMEM medium. Duplicate confluent Caco-2 cell monolayers were then inoculated with an average 1.6 × 10^7^ CFU/ ml (CI 95%: 1.1 × 10^7^–2.0 × 10^7^) of each LAB strain to be analyzed resulting in a multiplicity of infection (MOI) of 52.4, as determined by plating the LAB inoculum on MRS plates and counting the Caco-2 cells in a Neubauer chamber. Inoculated Caco-2 cell plates were returned to the water-jacketed incubator for 30 min to allow for LAB attachment. Caco-2 cell monolayers were washed once with 1 mL sterile phosphate-buffered saline (PBS, Thermo Scientific, Rockford, IL, United States) to remove non- or loosely adherent bacteria. Caco-2 cells were then lysed by addition of 1 mL of ice-cold water to release adherent LAB bacteria. Appropriate serial dilutions were plated onto MRS plates, incubated and LAB were enumerated as detailed above. The attachment efficiency of each LAB strain was assayed in two biologically independent experiments each with two technical replicates. Attachment efficiency was calculated by dividing the average number of adherent bacterial cells (across biological and technical replicates) by the number of Caco-2 cells in each well.

### Caco-2 Cell Cytotoxicity Assay

The *in vitro* cytotoxicity of the top ranking LAB strains against Caco-2 cells was evaluated by using a CytoTox 96 non-radioactive cytotoxicity kit (Promega, Madison, WI, United States), which measures release of lactate dehydrogenase (LDH) upon Caco-2 cell lysis, following manufacturer’s recommendations. Briefly, Caco-2 cells were seeded on a 24-well plate and grown to confluence as described above. Caco-2 monolayers were inoculated with each LAB strain at a level of 1.6 × 10^7^ CFU/ml (CI 95%: 1.1 × 10^7^–2.0 × 10^7^) and cytotoxicity was evaluated after 24 h by measuring absorbance (release of LDH) in a microplate reader (Biotek Instruments, Winooski, VT, United States) at 490 nm. The cytotoxicity of each LAB strain was evaluated in two biologically independent experiments each containing two technical replicates. In each independent experiment, two un-inoculated Caco-2 monolayers were included and used as maximum lysis controls (according to the kit instructions) and two un-inoculated Caco-2 monolayers were included to determine the background absorbance associated with the medium. Background absorbance was averaged and subtracted from all individual observed values for Caco-2 wells inoculated with a LAB strain within each independent experiment. Percent cytotoxicity was expressed as the adjusted average absorbance value (subtracting background absorbance) for each LAB strain divided by the absorbance for the maximum lysis control.

### Antimicrobial Susceptibility Profiling

Antimicrobial susceptibility profiling was performed following the National Antimicrobial Resistance Monitoring System (NARMS) protocol (FDA, 2016). LAB strains were streaked onto TSA plates and incubated at 37°C for 24 h. A single colony of each LAB strain was selected and sub-streaked onto TSA plates containing 5% defibrinated sheep blood (HemoStat Laboratories, Dixon, CA, United States) and incubated at 37°C for 24–48 h. Antibiotic susceptibility was evaluated using the Sensititre^TM^ Gram-positive MIC plate assayed on the Sensititre^TM^ automated antimicrobial susceptibility system (Trek Diagnostic Systems, Westlake, Ohio) following manufacturer’s instructions. *Enterococcus faecalis* ATCC 29212 was used as a quality control organism. Thirteen antimicrobial agents, included in the MIC plate assayed, were evaluated in this study including: ampicillin (AMP), clindamycin (CLI), daptomycin (DAP), erythromycin (ERY), gentamicin (GEN), levofloxacin (LVX), linezolid (LZD), penicillin (PEN), rifampicin (RIF), synercid (SYN), tetracyclin (TET), trimethoprim/sulfamethohazole (SXT), and vancomycin (VAN). The Minimum Inhibitory Concentration (MIC) breakpoints for the antimicrobials tested were interpreted based on the Clinical and Laboratory Standards Institute (CLSI) (Clinical and Laboratory Standards Institute, 2017).

### Whole Genome Sequencing and Bioinformatics Analyses

A total of 28 novel LAB strains with antagonistic characteristics toward *L. mono*cytogenes, *E. coli* O157:H7 and *Salmonella* were selected for further genotypic characterization. LAB strains were cultivated in MRS broth as detailed above, genomic DNA (gDNA) was isolated and purified using the Invitrogen Purelink DNA Extraction kit (ThermoFisher Scientific, Waltham, MA, United States). Pure gDNA was quantified using a Qubit^®^ 2.0 Fluorometer (Life Technologies, CA, United States), and used for library preparation with the Nextera XT v2.0 kit (San Diego, CA, United States) as per manufacturer’s recommendations. DNA libraries were subjected to paired-end sequenced using the 2 × 250 basepair (bp) V2 sequencing kit on an Illumina MiSeq platform (Illumina Inc., United States). Raw reads were preprocessed and filtered using Trimmomatic version 0.36 (Bolger et al., 2014), which was followed by *de novo* assembly using SPAdes version 11 (Bankevich et al., 2012). Resultant scaffolds were annotated using Prokka v1.13 (Seemann, 2014) and 31 conserved amino acid coding sequences were identified through the AMPHORA2 pipeline (Wu and Scott, 2012). The 31 conserved amino acid coding sequences were aligned and a concatenated alignment was created to compare our novel LAB strains to a large background of other strains representing genera in the LAB order. Taxonomic identification for each of our LAB strains was determined based on the highest confidence gene set.

Bacteriocins, virulence factors, and potential AMR genes were identified by comparing genome untranslated gene sequences identified during genome annotation to the BAGEL3, Virulence Finder, ResFinder and PlasmidFinder databases, respectively (Zankari et al., 2012; van Heel et al., 2013; Joensen et al., 2014). Un-gapped alignments with higher than 95% identity and 95% query coverage were identified as positive for the virulence factors and AMR genes were used to confirm the presence of these genes. A phylogenetic tree was generated using the concatenated alignment in RAxML (Randomized Axelerated Maximum Likelihood) (Stamatakis, 2014) on the CIPRES science gateway (Miller et al., 2010).

Antimicrobial resistance-encoding genes identified by using the ResFinder v3.0 (Zankari et al., 2012) and PlasmidFinder v2.0 (Carattoli et al., 2014) pipelines from the Center for Genomic Epidemiology website^1^ were compared using BLASTn against the GenBank nucleotide database using default settings for sequence identification. A multiple genome alignment was created using Mauve software (v2.4.0) to compare plasmid sequences identified in strains L22, L24-A, and L25 against plasmid sequence data from *Enterococcus faecium* (accession number KJ645709).

### Confidence Interval Estimation and Statistical Analyses

Confidence intervals (95%) for attachment and cytotoxicity assay data were estimated using the mean and standard deviation of the ratio of bacterial cells to Caco-2 cells (attachment efficiency) and percent cytotoxicity (calculated by dividing adjusted average absorbance values by the maximum lysis control absorbance value in each experiment), respectively. Attachment efficiency and percent cytotoxicity values were analyzed using an ANOVA followed by the Bonferroni familywise error correction for multiple comparisons. Strain to strain comparisons were made to identify statistically significant differences using R coding language (R CoreTeam, 2017). The R packages: ggplot, phangorn, tidytree, ggtree, phylotools, and ape (Wickham, 2009; Schliep, 2011; Popescu et al., 2012; Revell, 2012; Yu et al., 2017) were used to describe the relationship between attachment efficiency, percent cytotoxicity and number of predicted bacteriocin and virulence genes.

## Results

### Agar-Well Diffusion Assay

Thirty-eight of the LAB strains showed an antagonistic effect against at least one of the pathogens tested (*Salmonella*, *E. coli* O157:H7 and *L. monocytogenes*), the remaining six did not inhibit or reduce the growth of the pathogens analyzed in this study. Thirty-seven of the LAB strains showed antimicrobial activity against *L. monocytogenes* with clear zones of inhibition ranging from 8.5 to 24 mm when overnight LAB cultures were used and from 6.5 to 21 mm when CFF was used. Twenty strains were antagonistic against *E. coli* O157:H7 with a maximum inhibition zone of 12 mm when L15 strain was used, and 18 were antagonistic against *Salmonella*, with a maximum zone of inhibition of 11.5 mm produced by strain L24-B. No zones of inhibition were observed for either *E. coli* O157:H7 or *Salmonella* when only CFF was used. Overall scores of inhibition summed across overnight culture and CFF for all three pathogens ranged from 33.5 to 45 mm for the top 20 LAB strains. Strain L20-B produced the highest ranking inhibition but was not inhibitory against *Salmonella* or *E. coli* O157:H7. Strain L28 was the highest ranking antagonistic strain that was effective against all three pathogens. Twelve of the top 20 LAB strains were originally isolated from a bovine source (i.e., cattle feces or raw meat), while the eight remaining isolates were isolated from fruits (*n* = 6) or vegetables (*n* = 2). Overall, increased antimicrobial activity was found when overnight cultures were used compared to CFF (Table 1).

**Table 1:** Antimicrobial activity of novel lactic acid bacteria strains against *Listeria monocytogenes, Salmonella*, and *Escherichia coli* O157:H7 sorted by rank of overall antagonistic activity across all pathogens.

			Zone of inhibition averaged from duplicate wells (mm)						
			*Salmonella*		*E. coli* O157:H7		*L. monocytogenes*		Overall score^b^	
Rank	Strain ID	Source	Culture	CFF^a^	Culture	CFF	Culture	CFF	Culture and CFF


1	L20-B	Bovine	0	0	0	0	24	21	45
2	J7	Grape	0	0	0	0	23	20.5	43.5
3	L24-B	Bovine	11.5	0	0	0	17	15	43.5
4	J25	Grape	0	0	0	0	22.5	20.5	43
5	L28	Ground beef	8	0	8	0	14	12.5	42.5
6	J14	Grape	0	0	0	0	23	18	41
7	L15	Bovine	9.5	0	12	0	10	9	40.5
8	J27	Grape	0	0	0	0	21.5	18.5	40
9	J43	Carrot	0	0	0	0	21.5	18.5	40
10	J16	Grape	0	0	0	0	22	17.5	39.5
11	J34	Grape	0	0	0	0	21.5	18	39.5
12	L3-A	Bovine	7	0	9	0	11	11	38
13	L14-C	Bovine	11	0	7.5	0	10	9	37.5
14	L14-B	Bovine	11	0	10.5	0	9	6	36.5
15	L5-B	Bovine	6	0	7	0	11.5	11	35.5
16	L2-A	Bovine	7	0	8	0	10.5	8.5	34
17	L5-A	Bovine	0	0	8	0	15	11	34
18	J19	Cabbage	0	0	0	0	17.5	16	33.5
19	L14-A	Bovine	9	0	8.5	0	9.5	6.5	33.5
20	L8-A	Bovine	0	0	0	0	20.5	13	33.5
21	L22	Bovine	6	0	7	0	10.5	7	30.5
22	L19	Bovine	6	0	0	0	14	10	30
23	L8-B	Bovine	0	0	7	0	10.5	11.5	29
24	L30	Bovine	0	0	0	0	14.5	14	28.5
25	L4-B	Bovine	6	0	8	0	14	0	28
26	L4-A	Bovine	7	0	6.5	0	11.5	0	25
27	L1	Bovine	7	0	0	0	9	8.5	24.5
28	L23-A	Bovine	7	0	6	0	11	0	24
29	L21	Bovine	0	0	7	0	9	7	23
30	L25	Bovine	6.5	0	6.5	0	9	0	22
31	L6-A	Bovine	0	0	7	0	10.5	0	17.5
32	L27-A	Bovine	0	0	6	0	9	0	15
33	L23-B	Bovine	6	0	0	0	8.5	0	14.5
34	L26	Bovine	0	0	6	0	8.5	0	14.5
35	L13-A	Bovine	6	0	8	0	0	0	14
36	L29	Bovine	0	0	0	0	10	0	10
37	L11	Bovine	0	0	0	0	9.5	0	9.5
38	L12	Bovine	0	0	0	0	9	0	9
39	L10	Bovine	0	0	0	0	0	0	0
40	L16	Bovine	0	0	0	0	0	0	0
41	L24-A	Bovine	0	0	0	0	0	0	0
42	L6-B	Bovine	0	0	0	0	0	0	0
43	L7	Bovine	0	0	0	0	0	0	0
44	L9	Bovine	0	0	0	0	0	0	0

*^a^“CFF” indicates cell free filtrate prepared by passing overnight lactic acid bacteria cultures through a 0.45 μM*.

^*b*^Overall score summed from zones of inhibition observed from overnight lactic acid bacteria culture and CFF across all three pathogens (i.e., *Salmonella*, *E. coli* O157:H7 and *L. monocytogenes*).

### Competitive Exclusion in Broth Culture

The antimicrobial activity of all LAB strains evaluated in this study is shown in Supplementary Table 1. Pathogen reductions were analyzed at 6, 12, and 24 h after co-inoculation with respect to un-inoculated control cultures; for *E. coli* O157:H7 and *Salmonella* highest reductions were observed 6 h after co-inoculation (2.03 and 2.53 log_10_ CFU/ml, respectively). At 12 h, highest reductions were 1.12 and 1.36 log_10_ CFU/ml for *E. coli* O157:H7 and *Salmonella*, respectively. Twenty-four hours after co-inoculation, 43 of the strains reduced *L. monocytogenes* by 0.12–9.06 log_10_ CFU/ml. Strains L28 and L20-B had the highest antimicrobial activity against *L. monocytogenes* with reductions of 9.06 and 6.96 log_10_ CFU/ml, respectively. Forty-one of the strains reduced *E. coli* O157:H7 by 0.02 to 0.84 log_10_ CFU/ml, where L4-B and L20-B achieved the highest reductions of 0.84 and 0.82 log_10_ CFU/ml, respectively. Thirty-two of the LAB strains reduced *Salmonella* by 0.05–0.94 log_10_ CFU/ml with L24-B and L20-B as the most antagonistic with reductions of 0.94 and 0.84 log_10_ CFU/ml, respectively. Greater antimicrobial effects were observed against *L. monocytogenes* compared with the other pathogens evaluated, notably strain L28 completely eliminated *L. monocytogenes* after 24 h after co-inoculation (Supplementary Table 1).

Pathogen reductions were summed up across time points and all three pathogens, and novel LAB strains were ranked based on their increased antimicrobial effect; strain L28 had the highest reduction across all pathogens tested in this study (Supplementary Table 1). Overall scores for agar-well diffusion and competitive exclusion assays were summed up to determine the top 20 LAB strains (within each assay), L20-B, L28, J7 ranked as the LAB strains with the greatest antagonistic activity. The top 20 strains from each assay (*n* = 28 strains collectively) were further characterized by cell culture assays, antimicrobial susceptibility and whole genome sequencing.

### Caco-2 Cell Attachment and Cytotoxicity Assays

**Figure 1:**
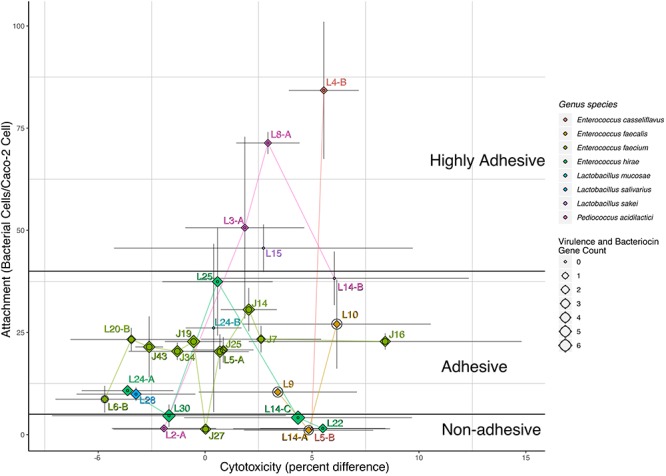
Attachment, cytotoxicity, virulence gene count, and bacteriocins. Labeled points of strain specific values for cytotoxicity and attachment are mapped to the *x*- and *y*-axis by bacterial cells/caco-2 cell and percent difference, respectively. Gray lines represent the confidence intervals for the mean estimate on either *x*- or *y*-axis. Points are colored and connected via lines of the same color based on each strain *genus* and *species.* Each labeled point is also represented by both a diamond and circle, the size of the diamond represents the number of bacteriocin genes, and the size of the circle represents the number of virulence-associated genes identified in the sequencing data. Black lines differentiate the adhesive potential as defined by Candela et al. (2005).

The ability of LAB strains to adhere to Caco-2 cells after 30 min was evaluated in this study. LAB attachment ranged from 4 to 84 bacterial cells/Caco-2 cell (Table 2). According to Candela et al. (2005) classification of microorganisms based on bacterial adhesive properties, five including L4-B, L8-A, L3-A, L15, and L2A were classified as highly adhesive, with > 40 bacterial cells/Caco-2 cells; 21 of the strains were classified as adhesive with 5–40 bacterial cells/Caco-2 cells and two were classified as non-adhesive with < 5 adherent bacterial cells/Caco-2 cells. The cytotoxic activity of our LAB strain panel was determined based on the release of the stable cytosolic enzyme LDH in the culture medium 24 h after bacterial inoculation. Percent cytotoxicity for the panel ranged from -5 to 8%, with L6B being the least cytotoxic and J16 the most cytotoxic (Table 2 and Figure 1).

**Table 2:** Novel lactic acid bacteria strains attachment to and cytotoxicity against Caco-2 cells.

Strain ID	Av. LAB cells/	Confidence	Av. Cytotoxicity	Confidence
	Caco-2 cell^a^	Interval (95%)	(%)^b^	Interval (95%)
L4-B	84.25	(67.46–101.04)	5.55	(4.10–7.00)
L8-A	71.37	(68.67–74.06)	2.94	(1.22–4.66)
L3-A	50.63	(28.37–72.89)	1.86	(-0.40–4.11)
L15	45.64	(39.84–51.44)	2.73	(-5.57–11.03)
L2-A	40.43	(19.44–61.42)	-1.93	(-2.35–(-1.50))
L14-B	38.25	(31.68–44.81)	6.04	(3.77–8.32)
L25	37.43	(24.23–50.63)	0.59	(-0.89–2.06)
L22	35.31	(22.67–47.96)	5.5	(4.43–6.58)
J14	30.59	(25.28–35.89)	2.04	(0.47–3.61)
L5-B	29.77	(20.36–39.18)	4.98	(0.88–9.08)
L10	27.05	(16.14–37.95)	6.16	(2.17–10.15)
L24-B	26.11	(5.52–46.69)	0.4	(-0.97–1.77)
J7	23.35	(20.11–26.60)	2.62	(-0.19–5.43)
L20-B	23.30	(20.48–26.12)	-3.45	(-6.11–(-0.79)
J16	22.80	(20.81–24.80)	8.42	(2.57–14.28)
J19	22.78	(21.57–23.99)	-0.54	(-2.02–0.95)
J27	22.09	(20.61–23.56)	0.02	(-5.20–5.24)
J43	21.46	(13.98–28.95)	-2.61	(-3.13–(-2.09)
J25	20.73	(17.59–23.87)	0.85	(-0.13–1.83)
J34	20.40	(18.26–22.54)	-1.3	(-3.78–1.18)
L5-A	20.31	(16.05–24.57)	0.69	(-0.73–2.12)
L14-A	12.33	(10.47–14.19)	4.84	(2.61–7.08)
L24-A	10.78	(9.79–11.76)	-3.63	(-5.57–(-1.68))
L9	10.42	(9.59–11.24)	3.4	(0.19–6.61)
L28	9.91	(8.24–11.57)	-3.23	(-5.13–(-1.33))
L6-B	8.67	(5.41–11.93)	-4.69	(-7.39–(-1.99))
L30	4.62	(1.88–7.37)	-1.69	(-7.08–(-3.69))
L14-C	4.14	(3.30–4.99)	4.35	(2.52–6.17)

*^a^Average (Av.) lactic acid bacterial cells/Caco-2 cell values were calculated from two biologically independent experiments each with technical replicates*.

^*b*^Average (Av.) cytotoxicity values were calculated from two biologically independent experiments each with technical replicates.

### Antimicrobial Susceptibility Profiling

All LAB isolates evaluated were susceptible to AMP, PEN, and LZD. Resistance to LVX was the most commonly found in our study (*n* = 17), followed by CLI (*n* = 9), VAN and SYN (each with *n* = 7), TET and SXT (each with *n* = 6), and RIF and GEN (each with *n* = 1). Intermediate resistance was mostly commonly observed for ERY, and DAP with 20 and 13 LAB isolates, respectively. Additionally, 7, 4, 3, and 2 LAB isolates exhibited intermediate resistance to GEN, TET, RIF, and SYN, respectively (Table 3). Three of the LAB isolates exhibited resistance to one antimicrobial agent (LVX); 16 were resistant to two antimicrobials, LVX-DAP was the most common AMR profile in this study with 9 of the isolates. Additionally, nine of all the LAB isolates in this study exhibited multidrug-resistance (MDR) with LVX-VAN-SXT as the most common AMR profile with 4 isolates, followed by DAP-TET-CLI-SYN with 3MDR isolates (Table 4).

**Table 3:** Summary of antimicrobial susceptibility pattern observed for novel lactic acid bacteria (LAB) strains.

Antimicrobial	Antimicrobial	Abbreviation	Susceptibility	No. of non-susceptible	
Class	agent		(%)^a^	isolates classified as	
				Intermediate	Resistant
Penicillin	Ampicilin	AMP	100.00 (28/28)	0	0
	Penicillin	PEN	100.00 (28/28)	0	0
Oxazolidinones	Linezolid	LZD	100.00 (28/28)	0	0
Rifamycin	Rifampicin	RIF	85.71 (24/28)	3	1
Folate pathway inhibitor	Tmp/Sxt	SXT	78.57 (22/28)	0	6
Glycopeptide	Vancomycin	VAN	75.00 (21/28)	0	7
Aminoglycoside	Gentamicin	GEN	71.43 (20/28)	7	1
Lincosamide	Clindamycin	CLI	67.86 (19/28)	0	9
Spectrogramin	Synercid	SYN	67.86 (19/28)	2	7
Tetracycline	Tetracycline	TET	64.29 (18/28)	4	6
Lipopeptide	Daptomycin	DAP	53.57 (15/28)	13	0
Fluoroquinolone	Levofloxacin	LVX	39.29 (11/28)	0	17
Macrolide	Erythromycin	ERY	28.57 (8/28)	20	0

*^a^Minimum Inhibitory Concentration (MIC) breakpoints were interpreted based on the Clinical and Laboratory Standards Institute (Clinical and Laboratory Standards Institute, 2017), where isolates were classified into categories of (i) susceptible or (ii) non-susceptible, which includes both resistant and intermediately resistant*.

**Table 4:** Description of antimicrobial resistance profiles of novel lactic acid bacteria (LAB) strains.

No. of antimicrobial resistance phenotypes observed^a^	Strain ID	Fluoroquinolone	Lipopeptide	Tetracycline	Glycopeptide	Folate pathway inhibitor	Lincosamide	Spectrogramin	Rifamycin	Aminoglycoside
	J16	LVX								
1	L5-B	LVX								
	L6-B	LVX								
	L28			TET	VAN					
	L20-B	LVX	DAP							
	J7	LVX	DAP							
	J14	LVX	DAP							
	J27	LVX	DAP							
	J43	LVX	DAP							
2	J34	LVX	DAP							
	J25	LVX	DAP							
	L5-A	LVX	DAP							
	J19	LVX	DAP							
	L30			TET			CLI			
	L10						CLI	SYN		
	L14-A						CLI	SYN		
	L9						CLI	SYN		
	L3-A				VAN	SXT				
	L8-A				VAN	SXT				
	L14-C	LVX			VAN	SXT				
	L15	LVX			VAN	SXT				
3	L14-B	LVX			VAN	SXT				
	L2-A	LVX			VAN	SXT				
	L4-B	LVX					CLI		RIF	
	L24-B		DAP	TET			CLI	SYN		
>3	L22		DAP	TET			CLI	SYN		
	L24-A		DAP	TET			CLI	SYN		
	L25		DAP	TET			CLI	SYN		GEN

*^a^DAP, daptomicyn; LVX, levofloxacin; TET, tetracycline; VAN, vancomycin; STX, trimethoprim-sulphamethoxazole; CLI, clindamycin; SYN, synercid; RIF, rifampicin; GEN, gentamicin*.

### Whole Genome Sequencing and Bioinformatics

**Figure 2:**
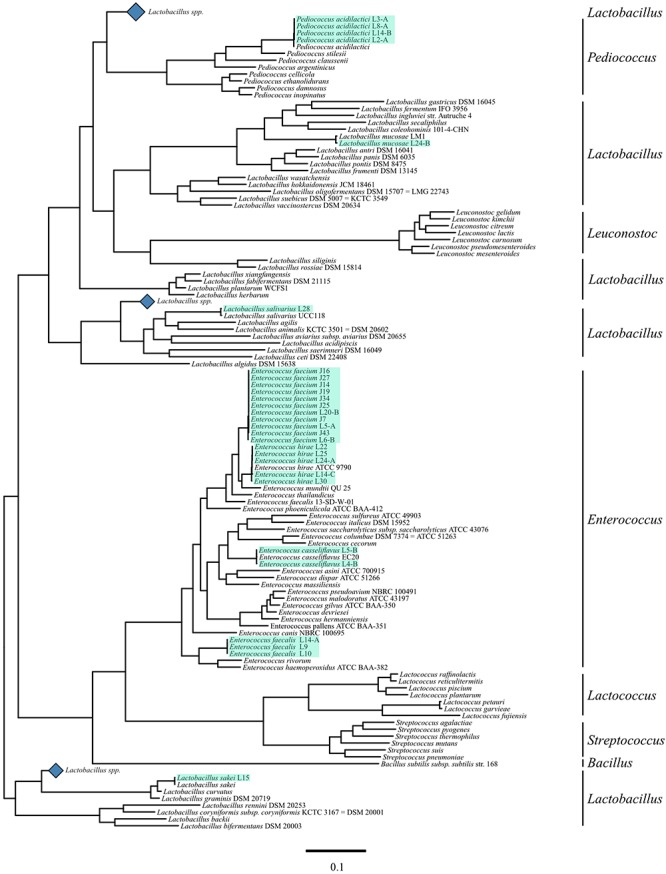
Phylogenetic tree of lactic acid bacteria (LAB) including novel LAB. Sequences of a selection of LAB genera, including sequenced strains, for each species available to be represented in a phylogenetic tree with mid-point root based on the 31 conserved gene amino acid sequences selected by the AMPHORA2 pipeline. The black bars on the right hand side represent genera markers. The blue diamonds represent collapsed clades of *Lactobacillus* species that did not contain strains phylogenetically related to the strains sequenced for this research. Light green highlights represent strains sequenced for this article.

Genotypic identification by WGS confirmed all as members of the LAB group; *Enterococcus* was the genus most frequently isolated accounting with 21 of the total LAB isolates. The seven remaining strains were identified as members of the *Pediococcus* and *Lactobacillus* genus with 4 and 3 isolates, respectively. *E. faecium* was the most common species identified in this study with 11 isolates, followed by *E. hirae*, and *P. acidilactici* with 5 and 4 LAB isolates, respectively (Table 5 and Figure 2).

**Table 5:** Whole genome sequencing of novel lactic acid bacteria (LAB) strains, bioinformatics analysis of sequences to identify putative bacteriocins and virulence factors.

Strain ID	Genus species	Putative bacteriocins	Virulence genes
L20-B	*Enterococcus faecium*	Enterocin B, enterolysin A	*efaAfm*
L28	*Lactobacillus salivarius*	Enterolysin A (2), salivaricin P	Non-identified
J7	*Enterococcus faecium*	EnterolysinA, lactacin F	*efaAfm*
J14	*Enterococcus faecium*	Enterocin B, lactacin F, enterocin L50A, enterolysin A	*efaAfm*
J27	*Enterococcus faecium*	Enterolysin A, enterocin B, lactacin F	*efaAfm*
J43	*Enterococcus faecium*	Lactacin F, enterocin (2), enteroloysin A	*efaAfm*
J16	*Enterococcus faecium*	Enterocin B, enterocin L50A, enterolysin A	*efaAfm*
J34	*Enterococcus faecium*	Enterolysin A, lactacin F, enterocin B, enterocin L50	*efaAfm*
J25	*Enterococcus faecium*	Enterolysin A, enterocin B	*efaAfm*
L5-A	*Enterococcus faecium*	Enterocin (2), enterolysin A, lactacin F	*efaAfm*
J19	*Enterococcus faecium*	Colicin V and bacteriocin-production cluster (4)	*efaAfm*
L14-C	*Enterococcus hirae*	Enterolysin A (3), closticin, carnocinCP52, sactipeptides	Non-identified
L14-A	*Enterococcus faecalis*	Lasso peptide	*camE, hylA, cOB1, efaAfs*,
L24-B	*Lactobacillus mucosae*	Non-identified	Non-identified
L3A	*Pediococcus acidilactici*	Pediocin	Non-identified
L15	*Lactobacillus sakei*	Non-identified	Non-identified
L5-B	*Enterococcus casseliflavus*	Non-identified	Non-identified
L14-B	*Pediococcus acidilactici*	Non-identified	Non-identified
L4-B	*Enterococcus casseliflavus*	Sactipeptides	Non-identified
L22	*Enterococcus hirae*	Enterolysin A (2)	Non-identified
L2-A	*Pediococcus acidilactici*	Pediocin	Non-identified
L8-A	*Pediococcus acidilactici*	Pediocin	Non-identified
L30	*Enterococcus hirae*	Enterolysin A (2), enterocin, closticin, sactipeptide	Non-identified
L25	*Enterococcus hirae*	Lanthipeptide, enterolysin A (2)	Non-identified
L10	*Enterococcus faecalis*	Lasso peptide	*camE, hylA, ebpA, ace, elrA*
L24-A	*Enterococcus hirae*	Enterolysin A (2), lanthipeptide class II	Non-identified
L9	*Enterococcus faecalis*	Lasso peptide	*camE, hylA, ebpA, ace, elrA*
L6-B	*Enterococcus faecium*	Lactacin F	acm, efaAfm

In this study between one and six putative bacteriocins were identified in 24 of the top LAB strains analyzed by genome comparison against the BActeriocin GEnome mining tool Database. Putative bacteriocins identified included enterolysin A, enterocin, lactacin F, sactipeptides, pediocin, closticin, lasso peptide, lanthipeptide, salivaricin, colicin V, and carnocinCP52. Enterolysin A was the most common being found in 15 of the isolates, followed by enterocins, and lactacin F found in 9, and 7 of the LAB strains, respectively (Table 6). No virulence factors were identified for 5 of the isolates, and virulence factors associated with cell adhesion were identified for 13 of the LAB isolates (Table 5).

**Table 6:** Description and summary of bacteriocins produced by novel lactic acid bacteria (LAB) strains.

LAB Strain ID	Genus species	Number of bacteriocins in each class											
		I					II					III	
		Lasso peptide	Lanthipeptide	Salivaricin	Sactipeptides	CarnocinCP52	Enterocin	Lactacin F	Pediocin	Closticin	Colicin V	Enterolysin	Total
L20-B	*Enterococcus faecium*	*–*	–	–	–	–	1	–	–	–	–	1	2
L28	*Lactobacillus salivarius*	*–*	–	1	–	–	–	–	–	–	–	2	3
J7	*Enterococcus faecium*	*–*	–	–	–	–	–	1	–	–	–	1	2
J14	*Enterococcus faecium*	*–*	–	–	–	–	2	1	–	–	–	1	4
J27	*Enterococcus faecium*	*–*	–	–	–	–	1	1	–	–	–	1	3
J43	*Enterococcus faecium*	*–*	–	–	–	–	2	1	–	–	–	1	4
J16	*Enterococcus faecium*	*–*	–	–	–	–	2	–	–	–	–	1	3
J34	*Enterococcus faecium*	*–*	–	–	–	–	2	–	–	–	–	1	3
J25	*Enterococcus faecium*	*–*	–	–	–	–	1	–	–	–	–	1	2
L5-A	*Enterococcus faecium*	*–*	–	–	–	–	2	1	–	–	–	1	4
J19	*Enterococcus faecium*	*–*	–	–	–	–	–	–	–	–	1	–	1
L14-C	*Enterococcus hirae*	*–*	–	–	1	1	–	–	–	1	–	3	6
L14-A	*Enterococcus faecalis*	1	–	–	–	–	–	–	–	–	–	–	1
L24-B	*Lactobacillus mucosae*	*–*	–	–	–	–	–	–	–	–	–	–	0
L3-A	*Pediococcus acidilactici*	*–*	–	–	–	–	–	–	1	–	–	–	1
L15	*Lactobacillus sakei*	*–*	–	–	–	–	–	–	–	–	–	–	0
L5-B	*Enterococcus casseliflavus*	*–*	–	–	–	–	–	–	–	–	–	–	0
L14-B	*Pediococcus acidilactici*	*–*	–	–	–	–	–	–	–	–	–	–	0
L4-B	*Enterococcus casseliflavus*	*–*	–	–	1	–	–	–	–	–	–	–	1
L22	*Enterococcus hirae*	*–*	–	–	–	–	–	–	–	–	–	2	2
L2-A	*Pediococcus acidilactici*	*–*	–	–	–	–	–	–	1	–	–	–	1
L8-A	*Pediococcus acidilactici*	*–*	–	–	–	–	–	–	1	–	–	–	1
L30	*Enterococcus hirae*	*–*	–	–	1	–	1	–	–	1	–	2	5
L25	*Enterococcus hirae*	*–*	1	–	–	–	–	–	–	–	–	2	3
L10	*Enterococcus faecalis*	1	–	–	–	–	–	–	–	–	–	–	1
L24-A	*Enterococcus hirae*	*–*	1	–	–	–	–	–	–	–	–	2	3
L9	*Enterococcus faecalis*	*–*	–	–	–	–	–	1	–	–	–	–	1
L6-B	*Enterococcus faecium*	–	–	–	-	–	–	1	–	–	–	–	1

### Molecular Characterization of Antimicrobial Resistance Genes

**Figure 3:**
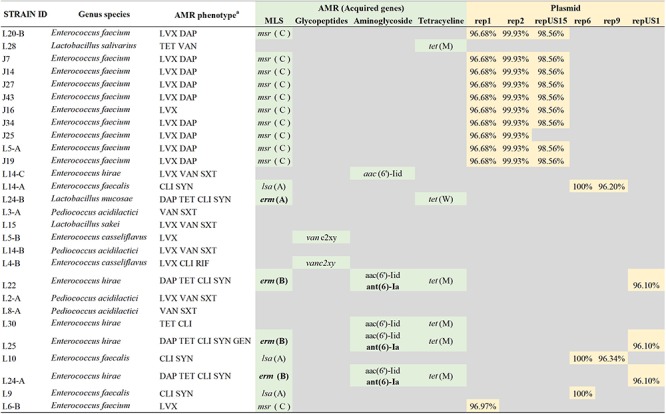
Comparison of antimicrobial resistance (AMR) phenotype and AMR genes identified and description of AMR gene. The most common AMR-encoding gene identified among the LAB strain set was *msr(C)* gene encoding for an ABC transporter associated with resistance to erythromycin, other macrolides, or streptrogramin B antibiotics (MS phenotype). The *msr(C)* was associated with the phenotypes showing resistant to less antimicrobials (LVX and LVX DAP profiles). *^a^*AMR phenotypes were determined using a Sensititre Gram-Positive MIC plate and using CLSI breakpoints. LVX, levofloxacin; DAP, daptomycin; TET, tetracycline; VAN, vancomycin; SXT, trimethoprim/sulfamethoxazole; CLI, clindamycin; SYN, synercid; RIF, rifampicin; GEN, gentamycin. Acquired antimicrobial genes were determined using the ResFinder v.3.1, and the plasmid incompatibility types were determined using PlasmidFinder v.2.0 pipeline. Percentage of similarity of the genes defining the incompatibility type with the reference sequences of the pipeline database. AMR genes indicated in bold were detected on the same contig that the incompatibility type genes, therefore most likely located on the plasmid.

A total of 23 of the investigated strains carried from one to four acquired genes associated with antimicrobial resistance. The most common AMR-encoding gene identified among the LAB strain set was *msr(C)* gene (*n* = 11) encoding for an ABC transporter associated with resistance to erythromycin, other macrolides, or streptrogramin B antibiotics. The *msr(C)* gene was always found alone and was associated with the phenotypes showing resistant to less antimicrobials (LVX and LVX DAP profiles). A total of four strains exhibited MDR phenotypic profiles (DAP TET CLI SYN, and DAP TET CLI SYN GEN), three of the these strains shared the same genotype: *erm(B)*, *aac(6′)-Iid*, *ant(6)-Ia* and *tet(M)*, encoding for a rRNA adenine N-6-methyltransferase, an aminoglycoside 6′-N-acetyltransferase, an aminoglycoside nucleotidyltransferase and a tetracycline resistance protein, TetM, involved in antibiotic target modification, respectively. Additionally, six different plasmid incompatibility types (rep1, rep2, repUS15, rep6, rep9, and repUS1) were characterized to determine if AMR-encoding genes were carried on the chromosome or plasmid. Seventeen strains harbored from one to three plasmids supporting the majority of AMR-genes were likely carried on plasmids. Further investigation elucidated that only plasmids belonging to the incompatibility type repUS1 were carrying antimicrobial genes (Figure 3). LAB strains, L22, L24-A, and L25, carried such plasmid harboring the *erm(B)* and *ant(6)-Ia* genes, involved in resistance to macrolides and aminoglycosides, respectively. BLASTn comparison showed that the repUS1 plasmids had 99% similarity out of 47, 48, and 68 of the total plasmid sequence registered under the accession number KJ645709, for the L22, L24-A, and L25 LAB strains, respectively.

## Discussion

Assessment of safety of potential probiotic strains is essential to be determined prior their use as feed additives. This assessment should include antimicrobial susceptibility to antibiotics of human and veterinary importance, attachment and cytotoxicity to intestinal epithelial cells, determination of presence of virulence factors and transmissible AMR genes. The aim of this study was to use a systematic approach to identify safe and effective novel LAB probiotic strains able to collectively control three important foodborne pathogens, including *Salmonella*, *E. coli* O157:H7 and *L. monocytogenes* along the food continuum through a combined phenotypic and genotypic characterization strategy.

Antagonistic activity was determined based on overall reductions or inhibitions across three important foodborne pathogens (i.e., *Salmonella*, *E. coli* O157:H7 and *L. monocytogenes*). Results showed that all candidate LAB strains evaluated were antagonistic to at least one of the pathogens tested. In agreement with other studies (Das et al., 2016; Abushelaibi et al., 2017), the antimicrobial effect was shown to be species and strain-dependent, with *E. faecium* L20-B as the top strain across pathogens and in both antagonistic assays; however, in agar well diffusion the strain was only effective against *L. monocytogenes*. Importantly, eight of our candidate LAB strains had an antagonistic effect against all the pathogens evaluated in this study. *L. salivarius* L28 was the top-ranking LAB strain that was effective against all three pathogens in both the agar well diffusion and competitive exclusion broth culture assays. Reductions found in this study were higher than those observed by other authors (Osuntoki et al., 2008; Angmo et al., 2016; Abushelaibi et al., 2017). Abushelaibi et al. (2017) observed zones of inhibition ranging from 0.1 to 2 mm for *Salmonella* and *E. coli* O157:H7 and >2.1 mm of inhibition for *L. monocytogenes* compared to the largest reductions of 12 mm for *E. coli* O157:H7, 11.5 mm for *Salmonella*, and 24 mm for *L. monocytogenes* observed in this study. Similar to our results, LAB isolates exhibited greater activity against *L. monocytogenes* compared to the other pathogens analyzed (Abushelaibi et al., 2017). Angmo et al. (2016) characterized LAB probiotic strains isolated from fermented foods, they also observed larger antagonistic activity of LAB isolates against Gram-positive bacteria including *L. monocytogenes* and weaker to medium activity against Gram-negative bacteria such as *E. coli.*

In this study, no zones of inhibition were observed for *Salmonella* and *E. coli* O157:H7 when using LAB CFF. *L. monocytogenes* was inhibited by overnight cultures of candidate LAB probiotic strains and by their CFF. *E. faecium* L20-B caused the highest inhibition; *E. faecium* strains are known by their anti-listerial activity, this antagonistic action is associated with the production of bacteriocins, such as enterocins (Corr et al., 2007; Kasra-Kermanshahi and Mobarak-Qamsari, 2015).

In the competitive exclusion assay, greater inhibition was also observed for *L. monocytogenes.* Notably, *Lactobacillus salivarius* L28 inhibited *L. monocytogenes* completely after 24 h of co-culture. Inhibition was highly probiotic strain dependent, for *Salmonella* and *E. coli* O157:H7 higher antagonistic effects were observed after 6 h of co-culture. As time increased, the antagonistic effect decreased, possibly by adaptation of the pathogens to the decrease in pH and/or the presence of antimicrobial compounds in the media. For *L. monocytogenes* the effect was different: as time increased, improved reductions were observed. It is important to highlight that all LAB strains tested remained at high concentrations (10^9^ CFU/ml) in the co-culture assay throughout the 24 h incubation period.

*Enterococcus* was the most prevalent genus identified among our strain collection. *Enterococcus* strains from bovine and produce sources ranked among the top ten LAB strains, and were antagonistic mainly against *L. monocytogenes*. *Enterococcus* strains are ubiquitous in nature and are known by their bacteriocinogenic effect (Marekova et al., 2003; Sabia et al., 2004). Bacteriocins are ribosomally synthesized antimicrobial peptides able to inhibit closely related or non-related bacterial strains (Yang et al., 2014; Alvarez-Sieiro et al., 2016). Their bactericidal mechanism is primarily directed toward the receptor-binding located on bacterial surface, and also by causing cell membrane permeabilization (Yang et al., 2014). The anti-listerial activity of our LAB strains could have been associated with the production of bacteriocins. In fact, 24 of the strains had between one and six putative bacteriocins. Class II (unmodified peptides with 30–60 amino acids and a size < 10 kDa), and class III (heat unstable large proteins with a molecular weight > 30 kDa) bacteriocins were the most commonly identified in *E. faecium* strains with enterocins, and enterolysins being the most predominant. Bacteriocin-producing bacteria target cytoplasmic membrane, in Gram-negative bacteria the presence of a lipopolysaccharide (LPS) layer confers protection against the bactericidal effects of bacteriocins (Alvarez-Sieiro et al., 2016). However, Gram-negative bacteria with outer membranes that have been compromised due to sub-lethal stresses (i.e., heating, freezing) could be killed by membrane permeabilization (Bromberg et al., 2004). In Gram-positive bacteria, the lack of this protective layer make them more sensitive to these antimicrobial compounds. *Salmonella* and *E. coli* were reduced by our candidate LAB probiotic panel by 0.1–0.9, and 0.02–0.8 log_10_ CFU/ml, respectively. It has been suggested that the antagonistic effect of some probiotic strains, including *Lactobacillus*, against *Salmonella* and *E. coli* O157:H7 is primarily due to the production of organic acids, mainly lactic and acetic acids (De Keersmaecker et al., 2006; Makras et al., 2006). Organic acids act by permeabilizing the outer membrane, allowing antimicrobial compounds to pass through and exert an antagonistic effect (Alakomi et al., 2000).

The use of probiotics strains is a natural alternative to reducing the use of antibiotics for growth promotion in animal agriculture and in human medicine and, possibly, the rapid emergence of AMR pathogens (Imperial and Ibana, 2016). For this reason, it is imperative to evaluate the AMR profile of candidate probiotic strains, where special consideration should be taken to separate intrinsic (i.e., from point mutations) from acquired resistance (i.e., from transfer of AMR genes and plasmids) (Sanders et al., 2010; Imperial and Ibana, 2016). A great variability in antimicrobial susceptibility was observed among the strains tested here. All LAB strains were susceptible to penicillin (ampicillin and penicillin), and oxazolidinone (linezolid) antimicrobial classes. These results correlate with the fact that no genes for resistance against these antimicrobials were identified here. Our observations are in agreement with those of Abushelaibi et al. (2017), with most of their probiotic isolates being susceptible to ampicillin, and penicillin.

It is well known that LAB strains have intrinsic resistance to vancomycin and beta-lactam (Tejero-Sariñena et al., 2012; Varankovich et al., 2015). Resistance to levofloxacin was the most common AMR phenotype observed here. No acquired genes associated with levofloxacin-resistance were identified. Levofloxacin is a second-generation fluoroquinolone, where resistance is related to a point mutation(s) in one or more genes encoding the type II topoisomerases (*gyr*A, *gyr*B, *par*C, and *par*E) present in a chromosomal region known as the quinolone resistance-determining region (Redgrave et al., 2014). In *Enterococcus* species, resistance to levofloxacin has been associated with the presence of *eme*A gene (Jia et al., 2014), which was not identified in the *Enterococcus* isolates studied here. Resistance to lincosamides (clindamycin) was the second most common AMR phenotype, with most of the resistance isolates belonging to the *Enterococcus* genus: eight strains; resistance to lincosamides is conferred by a species-specific chromosomal gene, *lsa*(A), which encodes for an ABC transporter (Singh et al., 2002). Bacteria harboring the *lsa*(A) gene express the LS_A_ AMR phenotype with cross-resistance to lincosamides and spectrogramins (Cattoir and Leclercq, 2017), as observed in this study.

A high percentage of LAB strains demonstrated intermediate resistance to erythromycin, which correlated with the presence of the *msr* (C) gene, a species-specific chromosomal gene of *E. faecium* that encodes for an ABC transporter and efflux pump (Cattoir and Leclercq, 2017). The *msr* (C) gene confers also the MS antimicrobial phenotype (resistance to erythromycin and type B spectrogramins). Its inactivation has resulted in increased susceptibility of *E. faecium* to MSB antimicrobials (Reynolds and Cove, 2005). Nine of the LAB strains exhibited multi-drug resistance; to further probe the presence of transferable AMR-associated genes, analysis of all candidate LAB strain genomes was performed. Only three of the candidate LAB strains carried resistance-associated genes in a plasmidic region. The presence of AMR-associated genes in plasmidic regions or mobile elements is of concern due to their potential to be horizontally transferred (Broaders et al., 2013; Imperial and Ibana, 2016). These plasmid-containing strains were not among the top strains that showed antagonistic activity against foodborne pathogens and should not be considered as potential probiotic strains. These results support the safety of our top twenty selected LAB strains.

To be effective, bacteria in probiotic preparations should be able to adhere to the intestinal epithelium without causing cytotoxicity, to ensure longer permanence in the GIT (Piątek et al., 2012; García-Hernández et al., 2016). The ability of probiotic strains to adhere to epithelial cells improves their antagonistic action by allowing them to outcompete pathogens for receptors on epithelial cells (Corr et al., 2009). All LAB strains evaluated were able to attach to Caco-2 cells, with adhesion efficiencies varying among strains. Adhesion to epithelial cells is dose, matrix, and strain-dependent (Jensen et al., 2012). Candela et al. (2005) classified microorganisms based on their adhesive properties into three categories: (i) non-adhesive strains, when less than 5 cells adhere to Caco-2, (ii) adhesive strains, when the effectiveness of adhesion means 5–40 cells adhered to one Caco-2 cell, and (iii) highly adhesive strains, when the level of adhesion exceeds 40 cells per one epithelial cell (Candela et al., 2005). Based on this classification, 5 of our isolates were highly adhesive, 21 were adhesive, and 2 were non-adhesive. One important difference between the Candela et al. (2005) study and the current study was the amount of time allowed for interaction between bacterial and Caco-2 cells. We chose 30 min of incubation as this is sufficient for initial attachment, the 18 h incubation time used by Candela et al. (2005) could have allowed for subsequent bacterial growth. Attachment efficiencies of our LAB strains were higher than those observed by Jankowska et al. (2008); their LAB isolates, including *Lactobacillus* and *Lactococcus* strains, were able to adhere to Caco-2 cells in a range between 0.5 and 5 bacterial cells per one Caco-2 cell after four h of incubation.

Caco-2 cytotoxicity based on the amount of LDH released into the medium was low, ranging from -4.69 to 8.42, after 24 h of inoculation with LAB strains. These values were lower than those observed by Awaisheh and Ibrahim (2009), who analyzed two probiotic isolates, *L. acidophilus* LA102 and *L. casei* LC232. Our LAB strains might be used as feed additives without causing cytotoxicity of epithelial cells, but this is clearly something that needs further investigation.

Four groups of virulence-associated genes were detected; two groups in *E. faecium* and two in *E. faecalis*. The first group of *E. faecium* strains carried a single virulence-associated gene, *efaAfm* encoding for EfaA, an important component of cell adhesion and biofilm formation homologous to PsaA in *S. pneumoniae* (Lowe et al., 1995). The second group, carried two virulence-associated genes, *acm* and *efaAfm*. These genes are both involved in cell adhesion; *acm* has also been highly associated with clinical isolates from humans (Nallapareddy et al., 2008). The third and fourth groups of virulence-associated genes are contained in three strains of *E. faecalis*; these groups comprised seven unique virulence-associated genes including a hyaluronidase gene, *hylA*. Hyaluronidases are normally associated with cell lysis and degradation (Kayaoglu and Ørstavik, 2004). Of interest, the three *hylA-*containing strains L9, L14-A, and L10, did not show higher levels of cytotoxicity than other LAB strains tested here (Bonferroni corrected *p*-value < 0.05). Additional genes found in these two groups included sex pheromone-associated genes (*camE and cOB1*), biofilm and cell wall adhesion genes (*ebpA, efaA, and ace*), and genes involved in macrophage persistence (*elrA*) (Nakayama et al., 1995; Brinster et al., 2007;Fisher and Phillips, 2009; Woods et al., 2017).

Overall, the results demonstrate the ability of our selected LAB probiotic strains to inhibit *L. monocytogenes, Salmonella*, and *E. coli* O157:H7. The strains exhibit important features that could enhance their antagonistic action (no AMR-encoding genes in mobile elements, production of bacteriocins, ability to adhere to epithelial, low cytotoxicity percentages). *L. salivarius* L28 was the top-ranking strain that was effective against all three pathogens in both the agar well diffusion and competitive exclusion broth assays. *L. salivarius* L28 not only demonstrated adhesion to and low cytotoxicity against Caco-2 cells but also carried a low number of virulence and AMR genes making this strain a particularly good candidate for further evaluation to control foodborne pathogens in pre- and post-harvest applications.

## Author Contributions

DA, PC, JF, and MB performed all experiments in the study. DA, PC, and KK contributed to the bioinformatics analyses. GL, MMB, and KN conceived the study. DA, PC, and KN contributed to writing and editing the final version of the manuscript.

## Conflict of Interest Statement

MMB, GL, and KN have ownership in NexGen Innovations, LLC. NexGen Innovations, LLC, has licensed some of the strains described in this study through Texas Tech University for commercial development and application. The remaining authors declare that the research was conducted in the absence of any commercial or financial relationships that could be construed as a potential conflict of interest.

## Footnotes

1 https://cge.cbs.dtu.dk/services/
