# Supplementary material for: A Systematic Approach to Identify and Characterize the Effectiveness and Safety of Novel Probiotic Strains to Control Foodborne Pathogens
Source: Front Microbiol. 2019 May 17;10:1108. doi: 10.3389/fmicb.2019.01108 (PMC6533568; doi:10.3389/fmicb.2019.01108)
Supplement: Supplementary file 1 [file Table_1.DOCX]

**Supplementary Table 1.** Pathogen reduction (log_10_ CFU/ml) by novel lactic acid bacteria (LAB) strains co-cultivated on TSB supplemented with one g l^-1^ Tween 80

| ***Salmonella*** | | | | | ***E. coli* O157:H7** | | | | ***L. monocytogenes*** | | | | **Combined Score** | **Strain ID** | **Rank** |
| --- | --- | --- | --- | --- | --- | --- | --- | --- | --- | --- | --- | --- | --- | --- | --- |
| **(Log10 CFU/ml)** | | | | | **(Log10 CFU/ml)** | | | | **(Log10 CFU/ml)** | | | |  |  |  |
| **Strain ID** | **6h** | **12h** | **24h** | **All time points** | **6h** | **12h** | **24h** | **All time points** | **6h** | **12h** | **24h** | **All time points** |  |  |  |
| L28 | 0.448 | 0.45 | 0.24 | 1.14 | 0.370 | 0.62 | 0.46 | 1.45 | 0.39 | 9.14 | 9.06 | 18.59 | 21.18 | L28 | 1 |
| J27 | 0.624 | 0.75 | 0.67 | 2.04 | 0.732 | 0.41 | 0.27 | 1.41 | 4.37 | 6.71 | 6.60 | 17.68 | 21.13 | J27 | 2 |
| L20-B | -0.037 | 0.90 | 0.84 | 1.71 | 0.969 | 0.97 | 0.82 | 2.76 | 3.43 | 6.36 | 6.71 | 16.49 | 20.96 | L20-B | 3 |
| J7 | 0.897 | 0.55 | 0.35 | 1.79 | 1.130 | 0.54 | 0.38 | 2.05 | 4.31 | 6.15 | 6.27 | 16.73 | 20.57 | J7 | 4 |
| J43 | 1.08 | 0.25 | 0.05 | 1.38 | 1.060 | 0.41 | 0.26 | 1.73 | 3.75 | 6.72 | 6.96 | 17.44 | 20.55 | J43 | 5 |
| J14 | 0.624 | 0.36 | 0.69 | 1.67 | 0.834 | 0.54 | 0.56 | 1.93 | 3.82 | 6.66 | 6.33 | 16.80 | 20.40 | J14 | 6 |
| L5-A | -0.123 | 0.21 | 0.18 | 0.27 | 0.940 | 0.81 | 0.69 | 2.44 | 3.75 | 6.57 | 6.53 | 16.86 | 19.57 | L5-A | 7 |
| J19 | 1.305 | 0.55 | 0.35 | 2.20 | 1.141 | 0.41 | 0.29 | 1.84 | 4.31 | 6.01 | 4.99 | 15.31 | 19.35 | J19 | 8 |
| J16 | 0.101 | 0.31 | 0.61 | 1.02 | 0.477 | 0.63 | 0.56 | 1.66 | 3.61 | 6.57 | 6.25 | 16.43 | 19.11 | J16 | 9 |
| J34 | 0.955 | 0.29 | 0.09 | 1.33 | 0.887 | 0.62 | 0.48 | 1.99 | 2.89 | 5.83 | 6.39 | 15.11 | 18.43 | J34 | 10 |
| L9 | 0.288 | 0.75 | 0.81 | 1.84 | 1.477 | 1.12 | 0.74 | 3.34 | 1.91 | 3.96 | 2.63 | 8.51 | 13.69 | L9 | 11 |
| L14-A | 1.424 | 1.18 | -0.16 | 2.44 | 1.285 | 0.29 | 0.23 | 1.81 | 1.47 | 3.57 | 4.21 | 9.25 | 13.50 | L14-A | 12 |
| J25 | 0.749 | 0.42 | 0.84 | 2.01 | 1.025 | 0.62 | 0.48 | 2.12 | 1.14 | 3.33 | 4.80 | 9.27 | 13.40 | J25 | 13 |
| L4-B | 1.040 | 0.42 | 0.74 | 2.20 | 1.069 | 0.79 | 0.84 | 2.70 | 2.83 | 2.57 | 2.50 | 7.90 | 12.80 | L4-B | 14 |
| L10 | 0.448 | 0.68 | 0.68 | 1.81 | 0.130 | 0.66 | 0.54 | 1.33 | 2.75 | 3.38 | 3.16 | 9.29 | 12.43 | L10 | 15 |
| L6-B | 0.911 | 0.78 | 0.70 | 2.39 | -0.024 | 0.94 | 0.78 | 1.70 | 0.94 | 3.25 | 2.74 | 6.93 | 11.02 | L6-B | 16 |
| L25 | 0.473 | 0.59 | -0.06 | 1.01 | 0.732 | 0.44 | 0.32 | 1.49 | 4.10 | 2.27 | 1.98 | 8.35 | 10.85 | L25 | 17 |
| L24-A | 0.527 | 0.25 | 0.31 | 1.08 | 2.033 | 0.39 | 0.42 | 2.84 | 2.61 | 2.15 | 1.92 | 6.69 | 10.61 | L24-A | 18 |
| L14-C | 0.323 | 0.51 | -0.13 | 0.71 | -0.008 | 0.26 | 0.19 | 0.44 | 1.77 | 3.36 | 3.42 | 8.55 | 9.70 | L14-C | 19 |
| L30 | 2.527 | 0.94 | 0.05 | 3.51 | 0.188 | 0.66 | 0.16 | 1.01 | 0.96 | 3.29 | 0.88 | 5.13 | 9.65 | L30 | 20 |
| L7 | 0.159 | 0.31 | 0.16 | 0.63 | 0.992 | 0.18 | 0.12 | 1.30 | 1.91 | 2.86 | 2.46 | 7.23 | 9.16 | L7 | 21 |
| L11 | 0.198 | 0.55 | 0.60 | 1.35 | 0.255 | 0.05 | 0.02 | 0.32 | 0.78 | 3.02 | 3.63 | 7.44 | 9.11 | L11 | 22 |
| L29 | 0.749 | 0.55 | 0.54 | 1.84 | 0.270 | 0.25 | 0.19 | 0.71 | 0.31 | 4.06 | 2.12 | 6.49 | 9.04 | L29 | 23 |
| L5-B | 0.589 | 0.97 | 0.76 | 2.32 | 0.477 | 0.41 | 0.29 | 1.18 | -0.18 | 2.50 | 3.05 | 5.36 | 8.86 | L5-B | 24 |

**Supplementary Table S1.** Continued

| ***Salmonella*** | | | | | ***E. coli* O157:H7** | | | | ***L. monocytogenes*** | | | | **Combined Score** | **Strain ID** | **Rank** |
| --- | --- | --- | --- | --- | --- | --- | --- | --- | --- | --- | --- | --- | --- | --- | --- |
| **(Log10 CFU/ml)** | | | | | **(Log10 CFU/ml)** | | | | **(Log10 CFU/ml)** | | | |  |  |  |
| **Strain ID** | **6h** | **12h** | **24h** | **All time points** | **6h** | **12h** | **24h** | **All time points** | **6h** | **12h** | **24h** | **All time points** |  |  |  |
| L27-A | 1.090 | 0.59 | 0.24 | 1.92 | 0.969 | 0.34 | 0.19 | 1.49 | 0.48 | 2.71 | 1.46 | 4.65 | 8.06 | L27-A | 25 |
| L22 | -0.029 | 0.67 | 0.39 | 1.03 | 0.502 | 0.47 | 0.61 | 1.58 | 0.42 | 2.61 | 2.33 | 5.36 | 7.97 | L22 | 26 |
| L23-B | 1.101 | 0.31 | 0.07 | 1.48 | 0.893 | 0.54 | 0.51 | 1.94 | 0.29 | 1.77 | 1.84 | 3.89 | 7.31 | L23-B | 27 |
| L26 | 0.979 | 0.18 | 0.24 | 1.40 | 0.732 | 0.31 | 0.55 | 1.59 | 0.35 | 2.22 | 1.61 | 4.18 | 7.17 | L26 | 28 |
| L12 | 1.342 | 1.36 | -0.17 | 2.53 | 0.528 | 0.50 | -0.09 | 0.94 | 1.26 | 1.71 | 0.69 | 3.66 | 7.13 | L12 | 29 |
| L14-B | 0.013 | 0.45 | -0.03 | 0.43 | 0.099 | 0.23 | 0.23 | 0.57 | 0.04 | 1.47 | 2.80 | 4.31 | 5.31 | L14-B | 30 |
| L13-A | 1.050 | 0.98 | 0.35 | 2.38 | 0.389 | 0.18 | 0.39 | 0.96 | 0.04 | 0.11 | 0.12 | 0.27 | 3.60 | L13-A | 31 |
| L21 | 0.557 | -0.03 | -0.12 | 0.41 | -0.074 | 0.27 | 0.62 | 0.82 | -0.09 | 0.66 | 1.78 | 2.35 | 3.58 | L21 | 32 |
| L19 | 0.323 | 0.31 | 0.13 | 0.77 | 0.051 | 0.10 | 0.16 | 0.31 | -0.79 | 1.17 | 2.11 | 2.49 | 3.56 | L19 | 33 |
| L23-A | 0.241 | 0.51 | 0.43 | 1.19 | 0.089 | -0.06 | -0.20 | -0.17 | -0.79 | 1.17 | 2.11 | 2.49 | 3.51 | L23-A | 34 |
| L4-A | 0.769 | 0.01 | -0.07 | 0.71 | 1.051 | 0.47 | 0.12 | 1.64 | -1.02 | 0.99 | 1.01 | 0.98 | 3.33 | L4-A | 35 |
| L24-B | 0.241 | 1.14 | 0.94 | 2.33 | 0.352 | 0.25 | 0.10 | 0.71 | -0.52 | 0.87 | -0.09 | 0.27 | 3.31 | L24-B | 36 |
| L6-A | 0.288 | 0.13 | 0.24 | 0.66 | 1.317 | 0.34 | 0.39 | 2.04 | -1.02 | 1.03 | 0.42 | 0.44 | 3.14 | L6-A | 37 |
| L2-A | 0.795 | 0.19 | -0.18 | 0.81 | 0.618 | 0.36 | 0.39 | 1.36 | -0.90 | 1.00 | 0.58 | 0.68 | 2.85 | L2-A | 38 |
| L15 | -0.068 | 0.05 | -0.14 | -0.16 | 0.016 | 0.10 | -0.08 | 0.03 | 2.74 | 0.09 | 0.12 | 2.95 | 2.82 | L15 | 39 |
| L3-A | 1.090 | 0.00 | -0.20 | 0.89 | 0.919 | 0.23 | 0.05 | 1.20 | -0.63 | 0.77 | 0.54 | 0.68 | 2.77 | L3-A | 40 |
| L16 | 0.123 | 0.18 | 0.11 | 0.41 | 0.431 | 0.20 | 0.07 | 0.70 | 0.78 | 0.31 | 0.39 | 1.49 | 2.60 | L16 | 41 |
| L1 | 0.935 | 0.36 | 0.11 | 1.41 | 0.410 | 0.47 | 0.39 | 1.27 | -0.83 | 0.10 | 0.26 | -0.48 | 2.20 | L1 | 42 |
| L8-A | 0.323 | 0.02 | -0.18 | 0.17 | 0.954 | 0.34 | 0.07 | 1.36 | -0.74 | 1.07 | 0.28 | 0.61 | 2.14 | L8-A | 43 |
| L8-B | 0.226 | 0.06 | -0.14 | 0.14 | 0.875 | 0.06 | 0.19 | 1.12 | -1.04 | 1.01 | 0.63 | 0.60 | 1.86 | L8-B | 44 |
